# Perspective: Utilizing High Amylose Wheat Flour to Increase Dietary Fiber Intake of Children and Adolescents: A Health by Stealth Approach

**DOI:** 10.3389/fpubh.2022.817967

**Published:** 2022-03-31

**Authors:** Kathryn Harris, Francine Overcash, Damien Belobrajdic, Joanne Slavin

**Affiliations:** ^1^Bay State Milling, Quincy, MA, United States; ^2^Department of Food Science and Nutrition, University of Minnesota, St. Paul, MN, United States; ^3^Commonwealth Scientific and Industrial Research Organization (CSIRO), Canberra, ACT, Australia

**Keywords:** fiber, resistant starch, high amylose wheat, adolescents, children

## Abstract

Children and adolescents have consistently failed to meet recommended levels of dietary fiber consumption, thus making fiber a nutrient of concern. The importance of adequate dietary fiber intake to attain a healthy diet necessitates the identification of fiber-rich and readily consumed food sources by youth. Grain-based foods derived from whole grains represent a strategic initiative to increase dietary fiber consumption and is consistent with the American diet pattern. Increased intake of foods made from whole grains have been positively associated with improved health outcomes but are also less preferred among youth compared to refined grain products, which make up the majority of their carbohydrate intake. Advances in the commercialization and availability of high amylose wheat flour, a source of resistant starch fiber, provides an opportunity to remedy the suggested acceptability issues of whole grain products indicative of sensory factors, without compromising the amount of fiber ingested. Resistant starch fiber consumption has been associated with health benefits including improved blood sugar management, improved markers of digestive and gut health, increased satiety, and a reduced inflammatory response among adults. The limited studies that indicate fiber's direct benefit among youth are largely observational, thereby necessitating the need for more controlled trials for these age groups. Replacing traditional refined wheat flour with refined high amylose wheat flour has the unique ability to increase dietary fiber consumption without compromising desired sensory and finished product characteristics and thus can help increase dietary fiber consumption in children and adolescents who struggle to meet adequate intakes of fiber.

## Introduction

Children and adolescents have consistently failed to meet minimum levels of fiber intake, leading it to be an official nutrient of concern (1, 2). Current recommendations set forth in the 2020–2025 Dietary Guidelines for Americans are based on adequate intakes of fiber that range from 17 g/d (ages 4–8 years) to 31 g/d (ages 14–18 years) (3). The extant literature is peppered with epidemiological evidence that continuously shows alarmingly low intake levels for all ages of youth (≤ 18 years). One study reported that nine out of 10 children failed to meet the National Academy of Medicine's Dietary Reference Intake (DRI) recommendation for fiber while another found both children and adolescents only consume half of the DRI recommendation for fiber (4). This fiber gap is even more pronounced in low-income and minority children (5) and appears to widen in adolescence (6).

## Dietary Fiber in Children and Adolescents

The importance of fiber to dietary quality necessitates the identification of fiber-rich and readily consumed food sources. Given an estimated 60 and 93% of youth aged 1–18 years do not meet recommended intake levels of fruit and vegetables respectively (7), dietary research has justifiably focused on fruits and vegetables as fiber-rich food sources (8–10). However, fruits and vegetables contribute <30% of the overall dietary fiber intake in children and adolescents, whereas cereal grains and their food sources contribute over 50% (11). Youth consume more than the recommended daily amount of grains (11), thus demonstrating the opportunity to make dietary modifications that incorporate whole grains and/or novel high amylose grains as a means for increasing dietary fiber intake in children and adolescents. A concerted effort to improve intake of nutrient dense grain-based foods fits within the American diet pattern (12), which may be advantageous for children and adolescents who are notoriously more selective in their food choices (13–15).

The recently released 2020–2025 Dietary Guidelines for Americans recommend at least half of the daily grain intake should be whole grains (16). Unfortunately, children and adolescents have consistently failed to meet these recommended levels. A study from a large representative sample of the US population (NHANES), found almost 40% of children and adolescents do not consume any whole grains (oz eq/d) and only 2.9% of children/adolescents (ages 2–18 years) met the minimum recommended intake of three whole grain oz equivalent servings per day (11). These whole grain deficiencies persisted in other studies that examined continuous years (≥10 years) of NHANES data from ~15,000 children/adolescents (6, 17).

## High-Amylose Wheat

While it is recommended that children and adolescents consume 2–5 ounce-equivalents of whole grains per day, the majority of their carbohydrate intake comes from refined grains (3). Consumer preference for refined bread is often cited as a reason for the relatively low consumption of whole wheat breads (18). Wheat is an important cereal grain that is a sustainable source of energy and is a staple food in the diets of most individuals around the world, but the way it is processed often determines its associated health outcome (19). When whole wheat kernels are processed into refined wheat flour, the fiber-rich bran and phytonutrient-rich germ layer are removed, and the starchy endosperm is milled to a fine particle size and used as the base ingredient for a broad range of processed foods such as breads, pastas/noodles, and tortillas (20). Products made with refined wheat flour do contain small amounts of dietary fiber, but these products generally elicit a high glycemic load due to the readily available, high digestible starch content in refined wheat flour, and thus their consumption has been associated with a number of chronic diseases (21). Wheat starch typically contains >70% amylopectin, a highly branched and rapidly digestible polymer, and <30% amylose, a linear polymer that is less digestible in the human small intestine. To address the nutritional inadequacies of refined wheat flour, high amylose wheat (HAW) varieties have been developed to deliver >80% amylose (22). The substantial increase in amylose within the endosperm of the wheat kernel delivers a higher resistant starch (RS) content, which is a form of non-digestible starch (23). Because wheat flour is the primary ingredient in a broad range of commonly consumed cereal products, any improvements to the nutritional value of wheat, like those achieved with this novel wheat variety, could have a positive impact of public health.

An alternate approach to increasing the dietary fiber content of cereal foods is to use these novel HAW varieties that yield refined flours naturally high in RS. Important products of intestinal fermentation are short chain fatty acids, which help to decrease colonic pH and maintain a healthy bacterial population in the large bowel (24). RS fermentation specifically favors the production of butyrate which is the primary energy source for colonic epithelial cells and it has antineoplastic effects in the colon (25). Consumption of foods high in RS have been shown to lower postprandial glucose responses in comparison to conventional starch-based foods and a sustained high intake of RS improves insulin sensitivity through mechanisms believed to be both dependent and independent of the byproducts of bacterial fermentation (26–28). Additionally, RS consumption has been associated with a number of health benefits including improved markers of digestive and gut health, increased satiety, and a reduced inflammatory response (29, 30).

Emerging clinical trials are demonstrating the functional health effects of foods made from HAW (31). This includes a lowered glycemic response for bread, noodles and pasta when conventional wheat flour is replaced with HAW (32–34). In particular the HAW breads, irrespective of whether they were made with whole wheat or refined flour, had a glycemic response that was 39% less than that achieved with conventional wheat breads and an insulinemic and incretin responses that were 24–30% less for HAW breads than for conventional wheat breads (33). Furthermore, foods containing refined HAW, but not whole wheat HAW showed favorable changes in fecal metabolites and bacteria species indicative of greater carbohydrate and lower protein fermentation compared to conventional wheat (35). Importantly, substituting conventional wheat flour with HAW in commonly consumed foods such as bread and biscuits enabled adults to increase their fiber intake (from a low background level of 20 g/d above the recommended level of 37 g/d) which was then well tolerated by the study participants (35, 36). Although these clinical trials on HAW have only used a healthy adult population (32–35) similar improvements in metabolic and gastrointestinal endpoints are expected in other age groups, including children and adolescents.

## Dietary Fiber Studies

The impact of fiber on children and adolescents' health has relied heavily on observational studies, while studies that show fiber's direct health benefits are limited at best. As such, the aforementioned recommendations for fiber intake among youth are extrapolated from studies among adult populations that showed improved outcomes with higher dietary fiber intake, namely cardiovascular disease and colon cancer (37, 38). Intervention studies among youth are often hampered by ethical and practical challenges in study design and methodologies (4). Constipation may be one of the more researched morbidities affected by fiber among youth, in part due to its inverse associations with quality-of-life scores and academic performance (39–42). In general, the correlations between fiber and obesogenic conditions have resulted in inconsistent findings. For example, two large-scale cross-sectional studies of American and British children found increased fiber was associated with reduced risk for overweight/obesity and lower body fatness, respectively (43, 44). On the other hand, another study found a null association between dietary fiber and adiposity in a sample of overweight Latino adolescents aged 10–17 years (45). Increased fiber intake was associated with reduced prevalence of metabolic syndrome, a grouping of multiple cardiometabolic risk factors including hyperglycemia/insulin resistance, obesity, and dyslipidemia in a study of 12 continuous years of NHANES data consisting of over 16,000 13–18 year olds (46).

## Discussion

While policy leaders have continuously pushed for increased whole grain consumption due to the higher vitamin, mineral, phytoestrogen, antioxidant, and dietary fiber content compared to its refined grain counterpart (12), refined grains continue to be the major contributor of dietary carbohydrates due to sensory preferences (18, 20). Because the most commonly cited reason for not consuming whole grain products is related to sensory attributes, it is important to ensure that the replacement of conventional refined flour with high amylose flour does not impart any differences in finished product quality. While there are other cereal-based ingredients that are high in resistant starch made from bananas (green banana flour) and corn (Hi-Maize^®^) there are considerable limitations to the amount of these ingredients that can be added to conventional cereal-based foods. For instance, adding 15% Hi-maize^®^ to a bread recipe increased the RS content of the bread to 5% but the bread had a worse appearance to the control and had a lower volume, and a lighter crust (47). While the addition of green banana flour can increase the RS content of bread to upwards of 6%, significantly lower loaf volumes, higher bread hardness, and darker crumb and crust colors have been reported at inclusion levels as low as a 10% replacement of wheat flour (48, 49).

Unlike other isolated fiber ingredients in high in resistant starch, HAW can replace upwards of 50% of conventional wheat flour without any deleterious effects in bread quality despite a 6X increase in resistant starch (50). In a recently conducted study, flour tortillas made with a 50% replacement of common wheat flour with HAW delivered 2.5 times more dietary fiber and had significantly higher rollability and foldability scores compared to flour tortillas made with conventional wheat (*p* < 0.05 for both, unpublished) even after 9 weeks of storage, demonstrating that the inclusion of HAW in this commonly consumed processed cereal food improved two important aspects of product quality with no deleterious effects on finished product quality. Consistent with these findings, a trained sensory panel reported that despite delivering three times more fiber, pasta made with 50% HAW flour and 50% semolina had no discernable differences in flavor, aroma, or texture (hardness, cohesiveness) when compared to standard pasta made with 100% semolina (unpublished data).

The effective incorporation of HAW flour into commonly consumed foods (i.e., breads/rolls, tortillas, pasta) supports the potential incorporation into other wheat-flour based foods including commonly consumed snack foods (i.e., crackers, biscuits/cookies), cereals, and pastries without adversely affecting the taste attributes of these products. While most of the research conducted on HAW has been done using bread, tortillas, and pasta as the delivery mode, refined HAW flour can readily replace common refined wheat flour in any of these wheat-based products to deliver higher amounts of dietary fiber, see Table 1. Given the well-known understanding that children like what they know and eat what they like (51), HAW has the unique potential to vastly increase dietary fiber consumption without compromising the desired sensory and finished product characteristics in commonly consumed foods and thus can serve as a platform for increasing dietary fiber consumption in children and adolescents who struggle to meet adequate intakes of fiber.

**Table 1:** Potential wheat-based foods that can be made with HAW and the subsequent impact on their fiber level.

**Wheat-based food product category/examples**		**Estimated fiber increase vs. conventional counterpart**	**Proposed quantity of HAW used in food formulation**
Yeast-raised dough-based systems (includes sweet doughs)	Pan breads/rolls/bagels (commercial and artisan-style), pizza, pastries, donuts, cinnamon rolls, etc.	**2-5X** depending on reference amount of food product, serving size of food product, and HAW inclusion in formula	**As a percentage of the total flour in formula: 5–100%** depending on desired fiber target by manufacturer
Chemically-leavened dough-based systems	Tortillas, flatbreads, shelf-stable pizza crusts, snack crackers, etc.	**2-5X** depending on reference amount of food product, serving size of food product, and HAW inclusion in formula	
Chemically-leavened batter systems (includes high-ratio formulations)	Pancakes/waffles, quick-breads, cake donuts, cookies, cakes, muffins, pie crusts, etc.	**2-10X** depending on reference amount of food product, serving size of food product, and HAW inclusion in formula	
Low-moisture baked and/or dry goods	Pasta, Ready-to-eat (RTE) cereals, cereal bars, ice cream cones, etc.	**2-10X** depending on reference amount of food product, serving size of food product, and HAW inclusion in formula	
Other	Retail flour, cream-of-wheat, sauces, confectionary, etc.	**2-10X** depending on reference amount of food product, serving size of food product, and HAW inclusion in formula	

As HAW has great potential to increase a child's fiber consumption, it is important to address the cost disparity that often comes with healthier food choices. A recently conducted meta-analysis concluded that healthier versions of grains and snacks/sweets were more expensive per serving, with an increase in $0.03/serving and $0.12/serving respectively (52). One reason healthier versions of grains and sweets/snacks can be more expensive is because of the addition of higher cost ingredients needed to meet superior nutritional targets such as whole grains, fiber isolates, and protein isolates. In addition to the compromised product performance, the cost of these ingredients may be prohibitive for food manufacturers due to their increased cost and ingredient declaration. Other cereal-based ingredients high in resistant starch undergo various manufacturing processes (separation and/or chemical modification) which drive higher raw material costs compared to HAW which is manufactured similarly to conventional wheat flour and thus is lower in cost. It is estimated that replacement of wheat flour with HAW may not have the same impact on price as it is a flour substitute. Using pasta as an example, replacement of 25% of standard wheat flour with HAW will increase fiber content by 3-fold, yet only increase the manufacturing cost by ~$0.05/1 lb. box, or ~$0.006/serving. Conversely, a standard pasta containing an isolated fiber additive providing an equivalent increase in dietary fiber would result in a larger increase in the manufacturing cost ~$0.10/1 lb. box, or ~$0.01/serving. However, it is unknown how the increased cost per serving will translate to a consumer because different food manufacturers will have different pricing models based on desired margin targets and revenue growth. Additionally, the cost of raw materials (i.e., HAW, isolated fibers, isolated proteins, etc.) vary depending on manufacturers, processing requirements, crop years, and/or crop quality. The commercial availability of cereal-based foods that contain HAW is currently limited to bread, tortillas, dry pasta, fresh noodles, pizza/pizza bases, ice cream cones, and cookies, this is anticipated to expand dramatically as the production of HAW increases and mainstream food manufacturers replace conventional wheat flour in existing cereal-based foods with HAW.

Closing the fiber gap among children and adolescents will remain a critical public health concern until new strategies and/or food products succeed where whole wheat has fallen short. A panel of leaders and experts in fiber research and industry took the official position that grain-based foods represent a pragmatic and “small step strategy” to increase total dietary fiber with reduced risk of increasing energy intake (12). The promising, albeit limited, results from the trials of high-amylose wheat products in adults make for a strong case to conduct controlled trials in youth that test the hypothesis of its mass appeal to increase fiber intake and deliver improved health outcomes, all without sacrificing desired sensory characteristics and thus ensuring consumption among children and youth.

## Data Availability Statement

The original contributions presented in the study are included in the article/supplementary material, further inquiries can be directed to the corresponding author.

## Author Contributions

KH, FO, DB, and JS have contributed significantly to the writing and editing of this manuscript and have approved the final version of the manuscript.

## Funding

Bay State Milling, Inc. financially supports FO.

## Conflict of Interest

FO is employed by the University of Minnesota but is financially supported by Bay State Milling for her time in writing this Perspective Manuscript. KH is a Research Food Scientist employed by Bay State Milling. The remaining authors declare that the research was conducted in the absence of any commercial or financial relationships that could be construed as a potential conflict of interest.

## Publisher's Note

All claims expressed in this article are solely those of the authors and do not necessarily represent those of their affiliated organizations, or those of the publisher, the editors and the reviewers. Any product that may be evaluated in this article, or claim that may be made by its manufacturer, is not guaranteed or endorsed by the publisher.
